# Molecular Dynamics Assisted Mechanistic Study of Isoniazid-Resistance against *Mycobacterium tuberculosis* InhA

**DOI:** 10.1371/journal.pone.0144635

**Published:** 2015-12-14

**Authors:** Vivek Kumar, M. Elizabeth Sobhia

**Affiliations:** Department of Pharmacoinformatics, National Institute of Pharmaceutical Education and Research, Sector 67, S.A.S. Nagar- 160 062, Punjab, India; University of Minnesota, UNITED STATES

## Abstract

Examination of InhA mutants I16T, I21V, I47T, S94A, and I95P showed that direct and water mediated H-bond interactions between NADH and binding site residues reduced drastically. It allowed conformational flexibility to NADH, particularly at the pyrophosphate region, leading to weakening of its binding at dinucleotide binding site. The highly scattered distribution of pyrophosphate dihedral angles and chi1 side chain dihedral angles of corresponding active site residues therein confirmed weak bonding between InhA and NADH. The average direct and water mediated bridged H-bond interactions between NADH and mutants were observed weaker as compared to wild type. Further, estimated NADH binding free energy in mutants supported the observed weakening of InhA-NADH interactions. Similarly, per residue contribution to NADH binding was also found little less as compared to corresponding residues in wild type. This investigation clearly depicted and supported the effect of mutations on NADH binding and can be accounted for isoniazid resistance as suggested by previous biochemical and mutagenic studies. Further, structural analysis of InhA provided the crucial points to enhance the NADH binding affinity towards InhA mutants in the presence of direct InhA inhibitors to combat isoniazid drug resistance. This combination could be a potential alternative for treatment of drug resistant tuberculosis.

## Introduction

The clinical isolates of mycobacterium tuberculosis bacilli obtained from the isoniazid (INH)-resistant tuberculosis (TB) patients are observed to have mutations in the structural gene of InhA protein [1–4]. INH react with NADH to form INH-NAD adduct which binds into dinucleotide binding site (DBS) of InhA. INH only blocks the site of hydride transfers by interacting with NADH that causes to inhibit the reduction of fatty acyl substrate. This is the molecular mechanism which makes INH an indirect inhibitor of InhA. However, INH does not contribute in INH-NAD binding into DBS. Thus, the observed mutations at DBS only affect NADH binding which ultimately leads to diminished INH-NAD binding affinity [5–8]. NADH interacts with DBS through H-bonds with side chain of residues Asp64, Ser20 and Lys165, and main chain of residues Val65, Gly14, Ile194, and Ile21 [7]. In addition to this, Dessen *et al*. reported water molecules in the DBS of InhA to mediate H-bond interactions between NADH and binding site residues. One of the water molecules is observed near the pyrophosphate moiety of NADH which mediates H-bonds with residues Ser94, Gly14 and Ala22. However, mutation of residue Ser94 into Ala94 causes disruption of bonding pattern which is accounted for considerable changes in NADH conformation. It is consistent with biochemical and genetic studies carried out by Basso *et al*., where a higher dissociation constant (K_d_) for NADH is observed for mutants I16T, I21V, I47T, S94A, and I95P as compared to InhA WT [9]. Oliviera *et al*. also reported the enhanced K_d_ values for I21V, I47T and S94A with the help of kinetic study [6]. Schroeder *et al*. also demonstrated the enhanced NADH conformational flexibility in I16T and I21V mutants using MD simulations [10]. These experimental and computational studies emphasize the influence of mutations on NADH binding which leads to INH-resistance. However, mutants I47T, S94A, and I95P are not explored at atomic level for deducing the INH-resistance using computational approaches. In addition to this, all mutant residues are not characterized at thermodynamic ground to compare the reduced binding energy contribution with respect to wild type residues. Many theoretical studies have shown the significance of computational approach in characterizing the mutation and ligand driven structural changes in vital proteins and their complexes [11–13]. MD simulation is one of the computational approaches which can be used effectively in the thermodynamic assessment of mutated residues on ligand binding and conformational changes [14–17]. The reported experimental studies show that mutations do present and plays a critical role in INH-resistance. However, the reported molecular modeling studies do not cover all InhA mutants. In addition, these studies also do not characterize the mutations on energetic ground of NADH binding.

The present study characterizes the mechanism of INH-resistance with respect to structural changes and binding energy of NADH using MD simulations. A deep analysis may substantiate the functional role of mutations in INH-resistance that can provide crucial structural insights to improve the INH binding. In addition to this, these structural insights can be viewed with respect to direct InhA inhibitor (DII) binding site adjacent to the DBS. It may lead us to explore the application of DIIs to enhance the INH binding in InhA mutants.

## Materials and Methods

### Data Selection

The binary crystal structure of InhA WT and MTs consisting of NADH were considered for comparative MD simulations studies. The initial structural coordinates were obtained from protein data bank (PDB) for WT (pdb id-2AQ8, 1.92Å) and MTs: I21V (pdb id-2AQH, 2.01 Å), I47T (pdb id-2AQI, 2.20Å) and S94A (PDB ID-2AQK, 2.30Å) [18–20]. In the absence of I16T and I95P mutant crystal structures, respective mutant models were built from WT using MOE (Molecular Operating Environment) molecular modeling package [21].

#### System preparation for molecular dynamics simulations

Preparation of the topology and coordinate files for the all complexes was carried out using the selected InhA crystal structure complexes. The obtained structures may have missing bond order, connectivity, steric clashes or bad contacts with the neighboring residues. Therefore, the selected structures were corrected for atoms and bonds and energy minimize to potentially relax the structures. It resolved steric hindrance and clashes in the structures. FF99SB force field parameters were set for the protein using the AMBER (Assisted Model Building with Energy Refinement) LeaP module [22]. The parameters missing for the NADH were taken from the amber parameter database, university of Manchester [23, 24]. The prepared systems were solvated with TIP3P water model by creating an isometric water box, where distance of the box was set to 10Å from periphery of protein [25]. Molecular systems were neutralized through the AMBER LeaP module by the addition of necessary amount of counter ions (Na^+^) to construct the system in electrostatically preferred positions. The whole assembly was then saved as per the requirement of free energy calculations. It involved preparing the parameter and coordinate files for the complex, protein and the ligand without solvation. Further, the prepared topology and coordinate files of solvated complexes were used as input for sander module of the AMBER [26]. The optimization and relaxation of solvent and ions were performed by means of two energy minimization cycles using 1500 and 2000 steps. The initial 1000 steps of each minimization cycle were performed using steepest descent followed by conjugate gradient minimization for rest of the steps. In the first part of minimization, InhA-NADH complex was kept fixed to allow water and ion molecules to move, followed by the minimization of the whole system (water, ions and complex) in the second part. Heating was performed into six steps using a NVT ensemble for 120ps where InhA-NADH complex was restrained with a very small force constant of 5kcal/mol/Å^2^. The temperature was allowed to increase by 50K in each step till 300K. The system was further equilibrated under constant pressure at 300K for the period of 100ps without restrain on the complex. Final simulations i.e. production phase were performed for 100ns on NPT ensemble at 300K temperature and 1atm pressure. The step size of 2fs was kept for whole simulation study. Langevin thermostat and barostat were used for temperature and pressure coupling. SHAKE algorithm was applied to constrain all bonds containing hydrogen atoms [27]. Non-bonded cutoff was kept on 10Å and long range electrostatic interactions were treated by Particle Mesh Ewald method (PME) with fast Fourier transform grid spacing of approximately 0.1nm [28]. Trajectory snapshots were taken at each 100ps of the production phase, which were used for final analysis. The minimization and equilibration were performed by sander module of Amber11, while production simulation was performed using Pmemd program of AMBER running on NVIDIA Tesla C2050 GPU work station [29]. The production run was considered for the analysis which was carried out using the Ptraj module of the Amber11 and VMD [30, 31].

#### Root-mean-square deviation, B-factor analysis, dihedral and Chi1 angles

The rmsd, B-factor, dihedral and Chi1 angles were calculated using the ptraj analysis tool in the AMBER program. The rmsd is the measure of the average distance between the atoms (usually the backbone atoms) of superimposed protein structures [32]. The equation below illustrates the rmsd:

$$rmsd=\frac{1}{N}\sum_{i}^{N}\delta_{1}^{2}$$ (1)

where δ is the distance between N pairs of equivalent atoms.

To compare the flexibility of the structures, B-factor was used to calculate the mobility of the residues present in the SBL. It was calculated from the mean square fluctuations (msf) using the following equation:

$$B-factor=\left[\frac{8\pi **2}{3}\right]\left(msf\right)$$ (2)

B-factor was calculated using the Eq 2 for each residue and plotted against each residue [33]. Dihedral angles (Φ, Ψ) are calculated for backbone of protein while Chi1 (χ1) dihedral angle referred to side chain dihedral angle of the residue [34]. They are calculated as per the following equations

ϕ=C−N−Cα−C (3)

Ψ=N−Cα−C−N (4)

χ1=N−Cα−Cβ−Xγ (5)

Where, N and C atoms are from NH2 and COO groups respectively, directly bonded to Cα atom of residue while Cβ represents side chain carbon atom bonded to Cα. Xγ represents the carbon/non-carbon atom bonded to Cβ.

#### Free energy calculations

Binding free energy calculations were performed for all complex systems using MM-GBSA (Molecular Mechanics Generalized Born Surface Area) considering single trajectory approach. The various previous study find this approach useful in estimation of binding free energy[35, 36]. Bello *et al*. identified the single trajectory approach useful for characterizing the key residues involved in valproic dehydrogenation using MM-GBSA [37]. Similarly, in another study, Bello *et al*. explored the binding of four fatty acids with β-lactoglobulin using the single trajectory approach and MM-GBSA method [38]. These cases depict the importance of the single trajectory approach in calculating the binding free energy using MM-GBSA. Binding free energy calculations were performed on production phase considering last 20ns of 100ns run using the Born implicit solvent model of 2 (igb = 2).

$$\Delta G_{binding}=G_{Complex}-G_{\mathrm{Pr}otein}-G_{Ligand}$$ (6)

The calculations include various energy components consisting of molecular mechanical energy (E_MM_) and polar contribution (G_GB_) towards solvation energy calculated by generalized Born (GB) method respectively (Eq 7). G_SA_ is the contribution from nonpolar terms towards solvation energy, and TS is the entropic contribution of the inhibitor. E_MM_ was obtained by summing contributions from, electrostatic energy (E_ele_), VDW energy (E_vdw_), and internal energy including bond, angle, and torsional angle energy (E_int_) using the same force field as that of MD simulations (Eq 8).

$$G=E_{MM}+G_{GB}+G_{SA}-TS$$ (7)

$$E_{MM}=E_{vdW}+E_{ele}+E_{\mathrm{int}}$$ (8)

Further, effective binding free energy was calculated using the frames obtained in every 5ns of 100ns simulations. The average of binding free energy was estimated for each 5 ns and referred as effective binding free energy

#### Energy decomposition analysis

Free energy was decomposed to estimate the contribution of each residue in the ligand binding process and was performed by using MM-GBSA method [39]. Energy of each residue–ligand interaction is given by following equation:

$$\Delta G_{residue-ligand}=\Delta E_{ele}+\Delta E_{vdW}+\Delta G_{GB}+\Delta G_{SA}$$ (9)

Where, ΔG_GB_ is the polar group contribution to the solvation free energy calculated using GB model. ΔG_SA_ is the non-polar group contribution to the solvation free energy calculated using ICOSA method. Similar to the binding free energy, the decomposition energy was also averaged over 200 frames taken at the interval of 100ps over the last 20ns of 100ns production run. In addition, average structures for comparison of trajectories were also obtained using the 200 frames over the last 20ns of 100ns simulations.

## Results and Discussion

### Structural features of InhA-NADH complex

**Fig 1:**
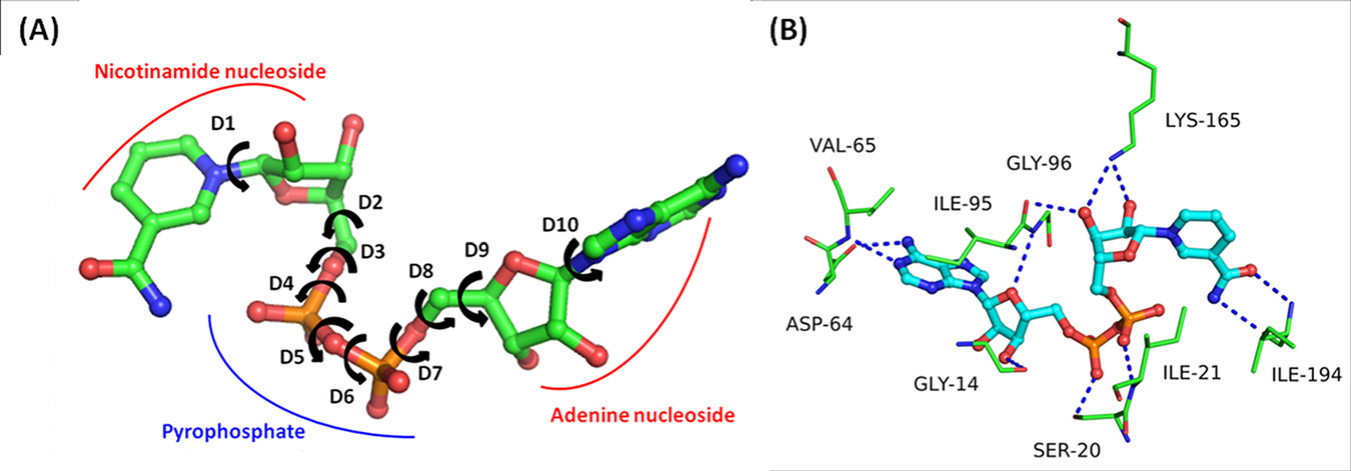
(A) NADH with various building blocks and dihedral angles. (B) NADH-InhA.

InhA catalyzes the fatty acyl substrate with the help of NADH co-factor. It is comprised of two nucleotide moieties coupled together through the phosphate groups making a pyrophosphate bridge. The nucleotide moieties of NADH consist of nitrogen base (one moiety consists of nicotinamide while other one consists of adenine), ribose sugar and phosphate group, while nitrogen base with ribose sugar is known as nucleoside (Fig 1A). The co-factor NADH contains ten single rotatable bonds (dihedral angle: D1-D10), and 8 of them are present in the pyrophosphate bridge contributing to its flexibility. NADH binds to the DBS through a network of eleven H-bond interactions with the active site residues. DBS is comprised of hydrophobic and polar residues. The residues Gly14, Asp64, Val65 and Gly96 are observed to make H-bond interactions with the adenine ring and adenine ribose moiety, while residues Ile95, Lys165 and Ile194 are found to interact with nicotinamide and ribose moiety of NADH. The residues Ser20 and Ile21 are observed to form H-bonds with the phosphate moieties of adenine and nicotinamide nucleotide respectively. In addition to these interactions, residue Phe41 is observed to make π-π interaction with the adenine ring of NADH. All mutants show H-bond interactions similar to the WT (Table 1). The details of H-bond donor and acceptor atoms with the distances are given in Table 1.

**Table 1:** Residues involved in H-bonding interactions to make InhA-NADH complex.

S.N.	H-bond donor	H-bond acceptor	H-bond distance (Å)					
			WT	I16T	I21V	I47T	S94A	I95P
1	O5(NADH)	CO (Gly14)	2.47	2.46	2.42	2.44	2.45	2.47
2	OH(Ser20)	O2(NADH)	2.01	2.01	2.00	2.04	2.05	2.02
3	NH(Ile21)	O9 (NADH)	1.81	1.80	1.96	1.90	1.89	1.81
4	H65(NADH)	OD1(Asp64)	2.05	2.05	1.98	2.02	2.00	2.05
5	NH(Val65)	N4 (NADH)	1.90	1.91	1.97	1.95	1.95	1.90
6	NH (Gly96)	O4(NADH)	2.30	2.31	2.29	2.30	2.31	2.30
7	NH_Z_ (Lys165)	O13(NADH)	2.03	2.02	1.98	2.00	2.01	2.03
8	NH_Z_(Lys165)	O12(NADH)	2.14	2.14	2.10	2.13	2.09	2.14
9	NH(Ile194)	O14 (NADH)	1.70	1.71	1.74	1.70	1.72	1.70
10	H86(NADH)	CO(Ile194)	2.31	2.31	2.33	2.29	2.30	2.31
11	OH(Thr196)	O9(NADH)	3.15	3.30	3.01	2.90	2.99	3.14
Total number of H-bond interactions			11	11	11	11	11	11

A distance cut-off of 3.30Å is considered for the H-bond with minimum cut-off of 140° for H-bond angle.

It is important to note that the residues that undergo mutation do not involve in direct H-bond interactions to NADH, except the Ile21 which does not show mutational effect as Val21 forms H-bond similar to WT. It may be due to the backbone atom of Ile21, which is involved in the H-bond interaction with NADH while the mutation only altered the side chain of the residue without affecting the backbone conformation. Further, these interactions are also aided by additional water mediated bridged H-bond interactions with NADH (Table 2). S94A is observed to make additional bridged H-bond interactions with NADH with the help of crystal waters WAT271 and WAT. In contrast to this, I16T, I47T, S94A, I95P and WT are not observed to form bridged H-bond interactions with these water molecules through Arg43 and, Ile95 and Lys165 through WAT396 and WAT287 respectively.

**Table 2:** Water mediated bridged H-bonding in the InhA-NADH complex in WT and MTs.

SN.	Residues	WAT	NADH*	WT	I16T	I21V	I47T	S94A	I95P
1	Gly14	271	O3, O9	Y	Y	Y	Y	Y	Y
2	Ala22	271	O3, O9	Y	Y	Y	Y	Y	Y
3	Ser94	271	O3, O9	Y	Y	Y	Y	Y	Y
4	Leu15	272	O6	Y	Y	Y	Y	Y	Y
5	Phe41	272	O6	Y	Y	Y	Y	Y	Y
6	Gly14	282	N5	Y	Y	Y	Y	Y	Y
7	Thr17	285	O2	Y	Y	Y	Y	Y	Y
8	Ile95	287	O12	-	-	Y	-	-	-
9	Lys165	287	O12	-	-	Y	-	-	-
10	Met147	288	O12	Y	Y	Y	Y	Y	Y
11	Gln66	349	N3	Y	Y	Y	Y	Y	Y
12	Tyr158	352	O14	Y	Y	Y	Y	Y	Y
13	Thr196	380	O8	Y	Y	-	-	-	Y
14	Arg43	396	O6	-	-	Y	-	-	-

H-bond cut-off of 3.30Å is considered with a minimum cut-off of 140° for H-bond angle.

*atom of NADH

Y = bond present

In addition to this, I21V, I47T and S94A are not observed to make bridged H-bond interactions with WAT380 through Thr196. I21V is observed with maximum bridged H-bond interactions while I47T and S94A are observed with minimum bridged H-bond interactions. Overall, a minimum 19 interactions are observed between the active site residues of DBS and NADH in the studied structures including direct and bridged H-bond interactions with residues. Among all complexes, mutant I21V is observed to have 23 interactions between the residues and NADH. Moreover, it is clear from the analysis that the maximum interactions are found near the pyrophosphate moiety of NADH. These interactions are supposed to impart rigidity to NADH conformation. Also, it is necessary to stabilize the flexibility of pyrophosphate to make a stable InhA-NADH complex.

Apart from the InhA-NADH interactions analysis, the distance between nearest atoms of mutated residues and pyrophosphate moiety of NADH is estimated. Comparison of distances shows that residues Ile16, Ile21, Ser94 and Ile95 are located within the radius of 5Å around the pyrophosphate bridge while residue Ile47 is located within a distance >6Å from pyrophosphate bridge. It suggests that the residues present in the vicinity of pyrophosphate may directly influence InhA-NADH interaction due the mutation. However, Ile47 will not probably affect InhA-NADH binding due to the mutational effect. In addition to location from NADH, mutated residues are also analyzed with respect to their location in the secondary structure of the InhA. Mutations located in the secondary structure may induce some distortional changes in protein topology. The mutations I16T, S94A and I95P are observed in the loop regions, and thus, are not supposed to affect the secondary structures of the InhA. I21V is found in the helix region, however, it is observed to restore the bonding pattern similar to corresponding secondary structure in WT. Similarly, I47T is also found in the helix region and observed to restore the bonding pattern similar to WT. Thus, the observed mutations are not observed to bring the distortional changes in the secondary structure of InhA.

### Comparative molecular dynamics simulations

#### Root mean square deviation, B-factor and effective energy

**Fig 2:**
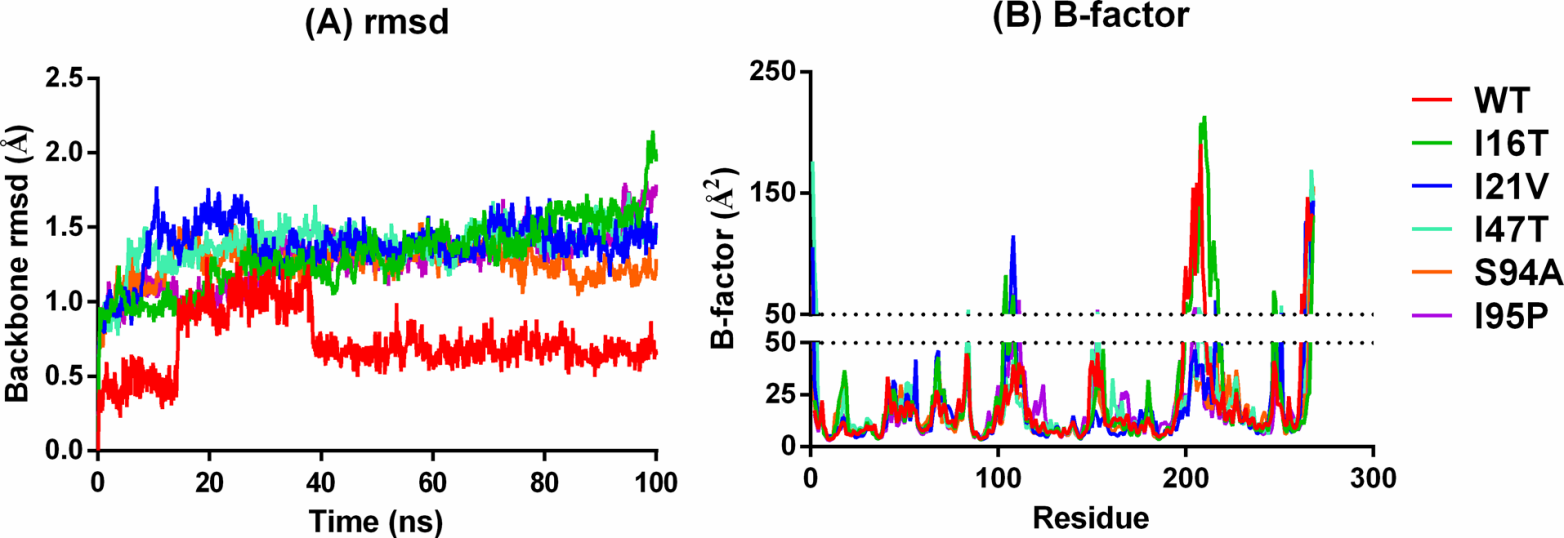
(A) InhA backbone rmsd and (B) B-factor of WT and MTs.

To obtain an estimate of the quality and convergence of the MD trajectory, the backbone root mean square deviation (rmsd) of InhA is calculated for the WT and MTs (Fig 2A). After a rapid increase during the first 40ns, the backbone rmsd of WT stabilized with an average rmsd of 0.72Å. The backbone rmsd of the MTs is also stabilized after 40ns of the simulation; however, it shows a higher average rmsd of ~1.2Å as compared to the WT. It indicates that enhanced flexibility of the mutant structures which may have occurred due to the distorted H-bonding pattern between InhA and NADH. In addition to this, the mutational effect is also expected for the disturbed InhA-NADH interactions leading to an increase in the fluctuation of binding residues. The order of the average rmsd for WT and MTs is observed as: WT (0.67Å) < S94A (1.28Å) < I95P (1.39Å) < I21V (1.41Å) < I47T (1.42Å) < I16T (1.44Å). This order demonstrates that InhA-NADH bonding pattern is drastically disturbed resulting in high fluctuations in backbone of MTs as compared to WT. Overall, these observations lead us to infer that there is a weakening of InhA-NADH interactions in the MTs due to mutations at the DBS. However, the average rmsd of the InhA backbone is observed below 0.8Å for the WT while it remains near to ~1.5Å for the MTs. It shows the convergence of trajectory and supports its use for further analysis.

The large differences between the backbone rmsd of MTs and WT can be rationalized on the basis of higher flexibility of loop regions estimated as B-factor. The major contribution to rmsd is shown by the residues 101–107 and 194–210 corresponding to A-loop and substrate binding loop (SBL) respectively (Fig 2B). It is important to note that these loops are the part of substrate binding pocket (SBP) and play a crucial role in substrate binding. Thus, the large humps are observed for SBL and A-loop in Fig 2B which is consistent with the necessity of these regions to undergo conformational changes upon binding of fatty acyl substrate. Apart from these regions, the overall B-factor for rest of the structures in MTs is observed similar to WT.

**Fig 3:**
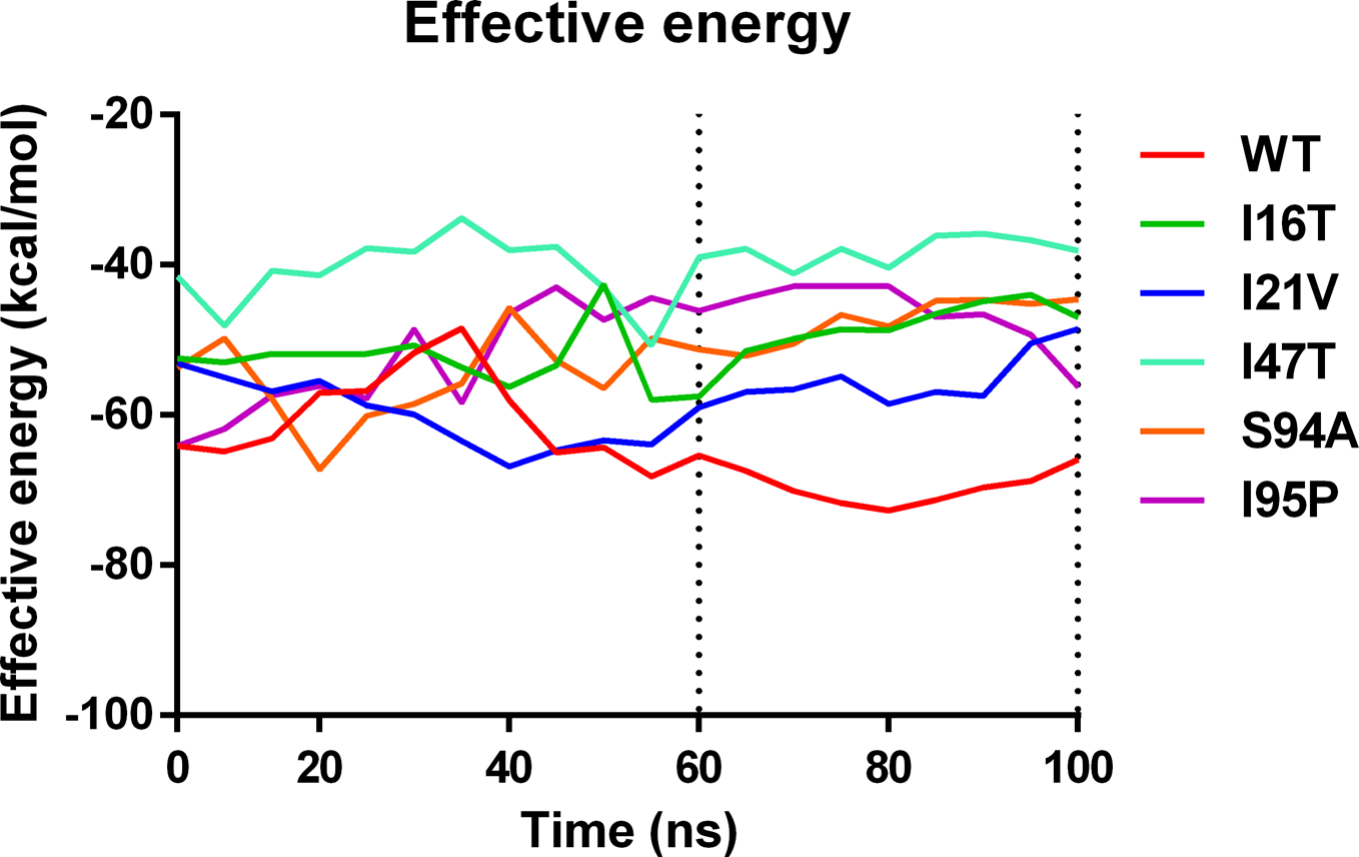
Effective average binding energy of complexes throughout the simulations.

The rmsd pattern provides sufficient information about time when the system reached equilibrium. However, as suggested by Bello *et al*., the effective binding free energies can also provide information about system convergences to support rmsd based convergence [40]. Fig 3 shows that all six systems shows fluctuation in average effective binding energy before 60ns. However, after 60ns of simulation trajectory shows a smooth convergence into a stable energy curve. This shows that all complexes achieve a stable effective energies after 60ns.

Finally, these observations indicate the enhanced flexibility of binding residues in DBS as a result of disturbed InhA-NADH interactions. It will lead to conformational changes in NADH and binding residues particularly mutated residues which can be analyzed by measuring dihedral angle for NADH and side chains of residues.

#### NADH dihedral angle distributions in WT and MTs

The variations in the rmsd of WT and MTs indicate a probability of weakening the InhA-NADH interactions due to the influence of the mutation at the DBS. It leads to the enhanced flexibility of NADH as well as binding site residues. The generated hypothesis from the earlier discussion is also consistent with the reported experimental study where mutations associated with the DBS lead to an increase in the NADH dissociation constant [20].

In addition to this, side chains of the mutated residues are small as compared to that in WT which may lead to a small gap between NADH and the residues in MTs structures. This small space may be a sufficient space for the conformational flexibility of NADH. A large gap between interacting atoms will also lead to weak interactions. In case of S94A, a large difference is observed in the length of side chain and polar nature of residues which may lead to a little large gap between interacting atoms of mutated residue and NADH. Consequently, it will increase the flexibility of NADH conformation. Similarly, in case of I95P, small side chain of mutated residues may also lead to more conformational freedom to NADH. I16T also undergoes the reduced side chain length and change in polarity which is supposed to affect InhA-NADH interactions. Although, residue Ile47 is located about >6Å away from the NADH binding site, change of Ile47 into Thr47 leads to change in polarity which may affect the microenvironment around NADH. Further, it may lead to enhanced conformational flexibility of NADH. Unlike other mutations, I21V does not undergo a change in amino acid polarity, however, reduced side chain length due to mutation is supposed to diminish the InhA-NADH interactions and enhance NADH conformational flexibility. Thus, it can be presumed from the discussions that a difference in side chains and polarity of residues upon mutation may lead to the conformational flexibility of the NADH. Conversely, these changes will also enhance the mobility of mutated residues. These conformational changes indicate the weakening of the InhA-NADH interactions and can be measured on the basis of dihedral angle calculations for NADH and mutated residues.

**Fig 4:**
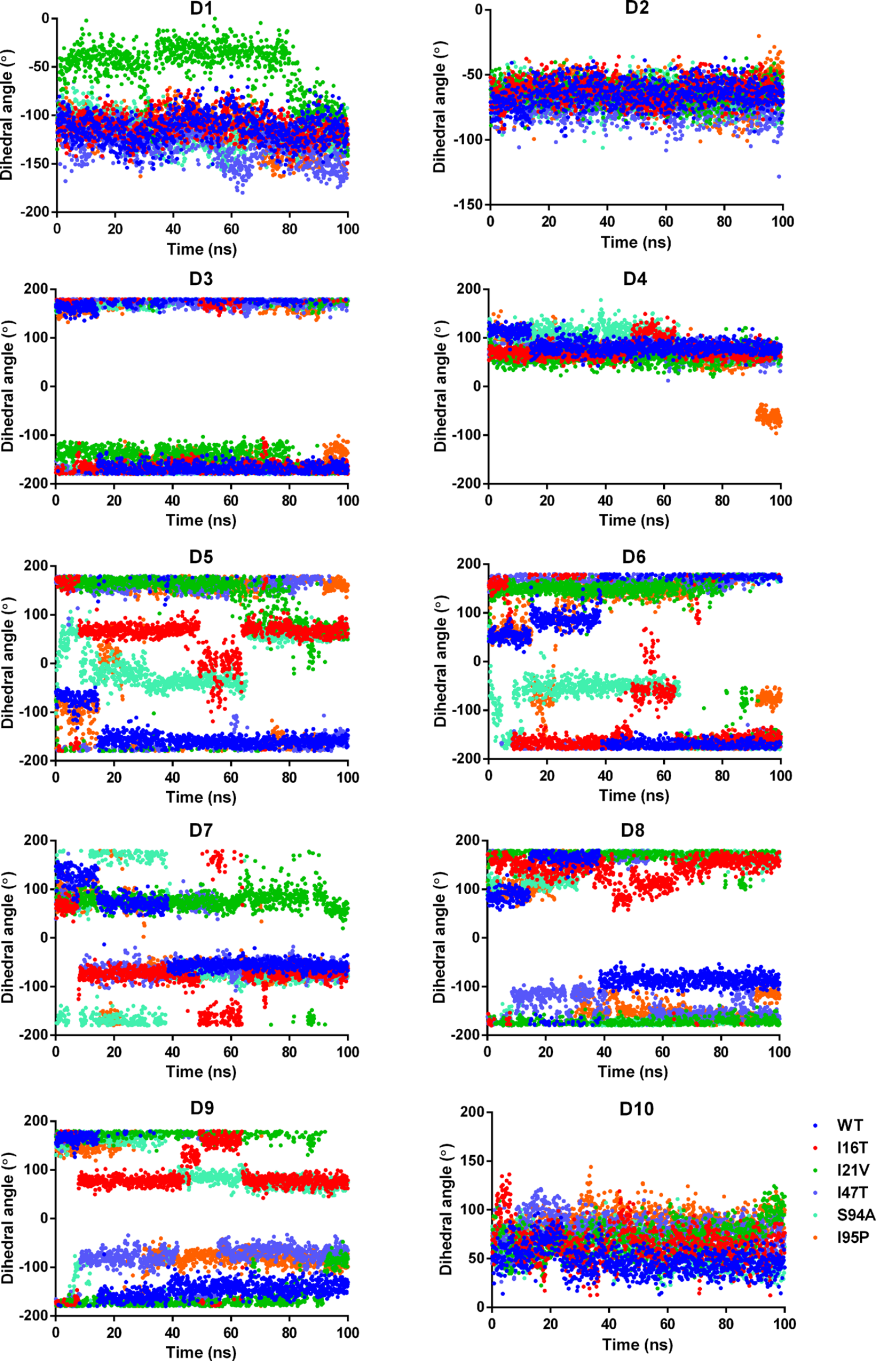
The dihedral angle distribution of NADH in WT and MTs.

**Fig 5:**
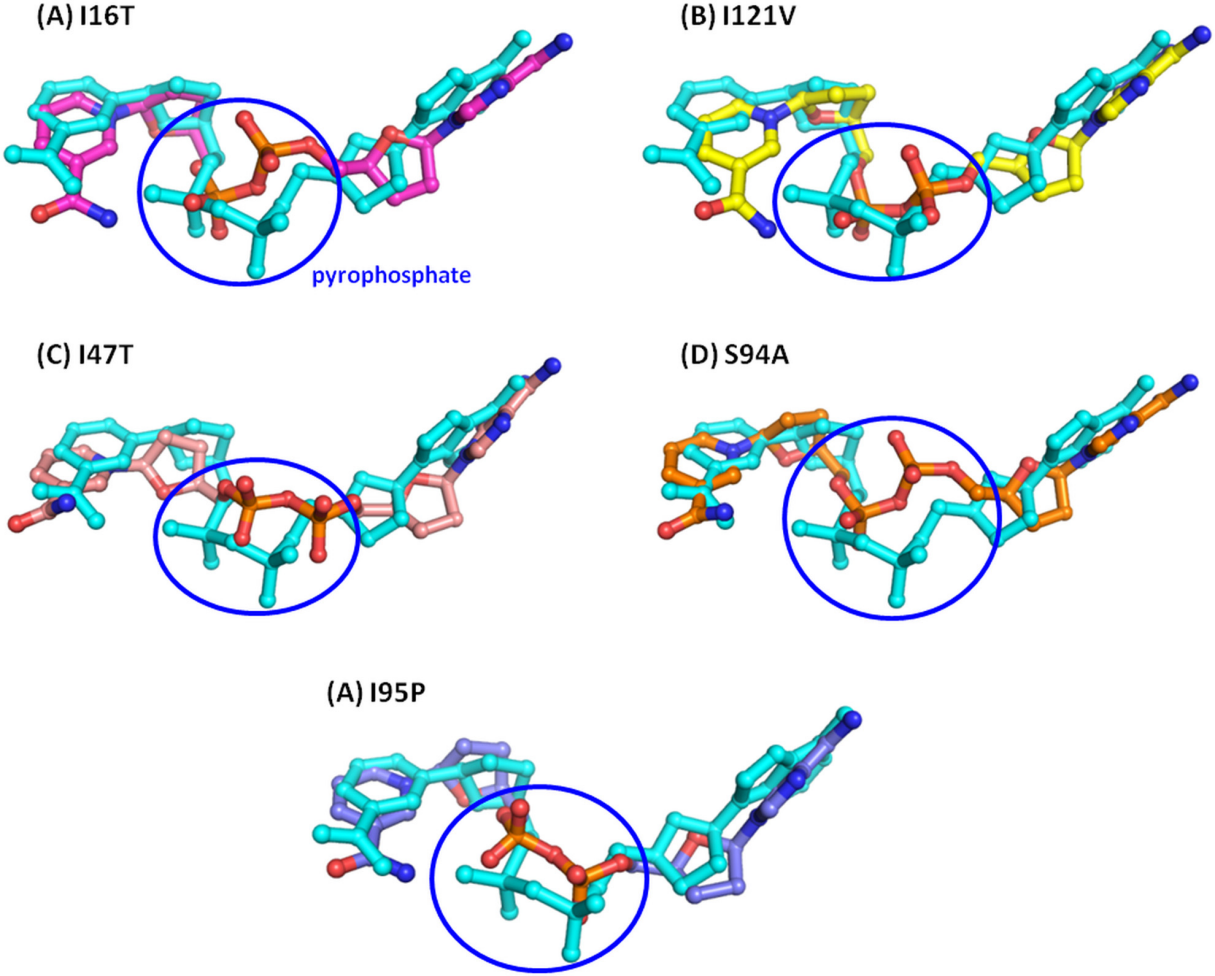
Comparison of NADH structure of WT (cyan) with MTs (colored).

Fig 4 shows the changes in the dihedral angles (D1 to D10) present in the NADH throughout the 100ns simulations of WT and MTs. The dihedral angles D1, D2, D3, D4, and D10 of NADH in the MTs are maintained at about the dihedral angle distribution of NADH in WT. However, rest of the dihedral angle in MTs varied significantly from the corresponding dihedral angle in the WT. It shows that adenine and nicotinamide moieties of NADH do not undergo major conformational changes and maintain their interactions with the nearby residues Asp64, Val65, Gly96, Lys165 and Ile194. Fig 5 shows the comparison between average NADH conformations from WT and MTs.

In case of I16T, comparison of NADH conformation shows a rmsd difference of 1.28Å from average NADH conformation from WT structure. The major differences are observed at the pyrophosphate region shown in blue colored circle in Fig 5 which makes H-bond interactions with Ser20 and Ile21. In addition to this, conformational differences are also observed for adenine and nicotinamide moiety due to variations in the pyrophosphate conformations. Similarly, NADH conformation in I95P shows a rmsd difference of 1.27Å from NADH conformation in WT. The large differences at pyrophosphate regions are observed in I17T and S94A where a rmsd difference of ~1.60 is estimated with respect to NADH conformation in WT. In case of I21V, a minimum rmsd difference of 0.90 is found with respect to NADH conformation in WT which depicts little better NADH binding in I21V as compared to other MTs. It clearly shows that the distribution of dihedral angles D5, D6, D7, D8 and D9 is highly scattered, which suggests sufficient conformational flexibility of the corresponding fragment in NADH i.e. pyrophosphate moiety. Moreover, enhanced flexibility of NADH is expected to diminish InhA-NADH interactions which will also increase flexibility of binding site residues.

#### Dihedral angle distribution of mutated and active site residues in WT and MTs

**Fig 6:**
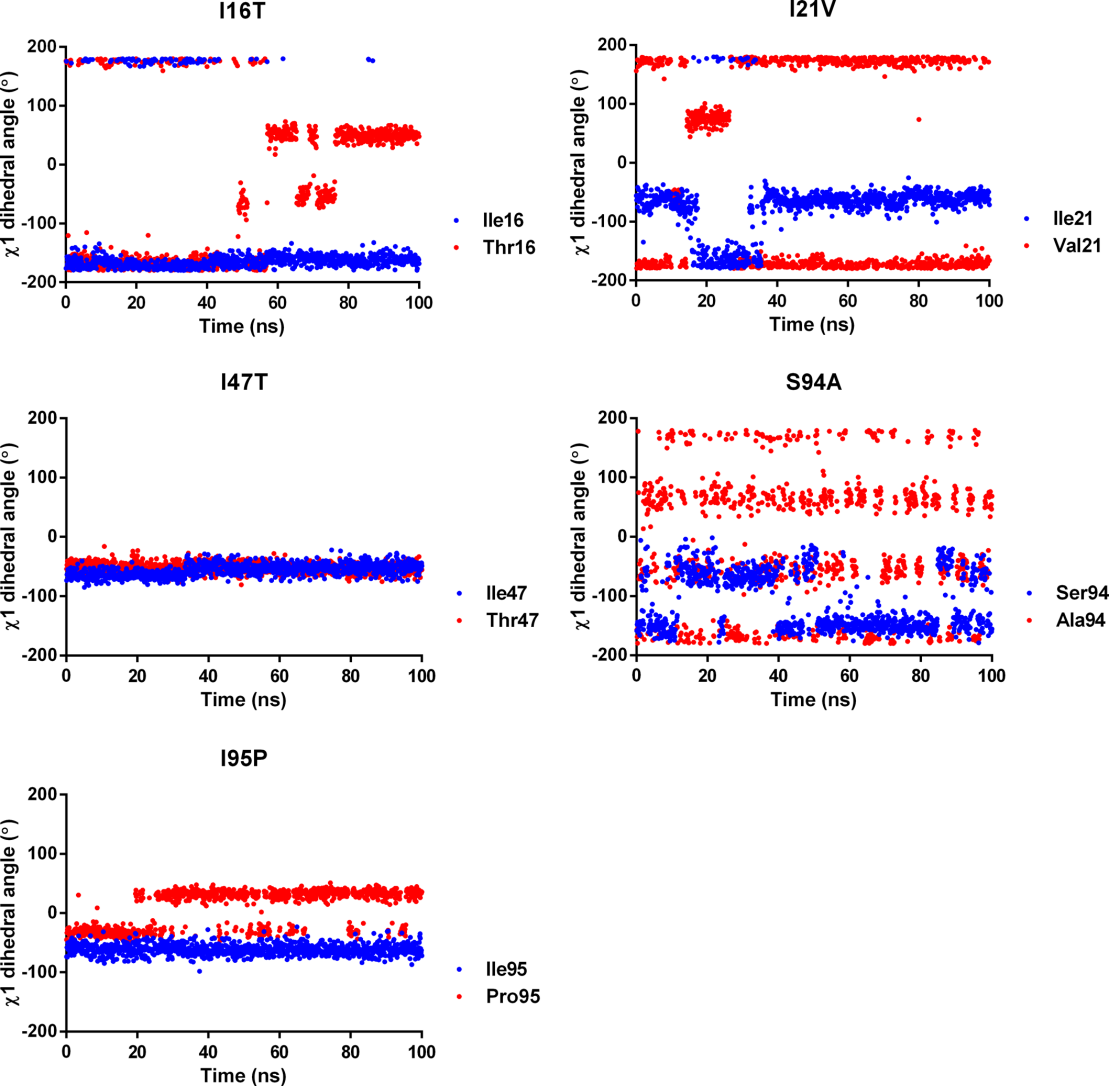
Dihedral angle distribution of mutated residues and respective wild type residues.

The enhanced flexibility of NADH conformation indicates weakening of NADH interactions with active site residues. Therefore, corresponding changes are also expected to occur in the side chain dihedral angle (χ1) of mutated residues involved in interactions with pyrophosphate moiety. The χ1 dihedral angle is estimated for the mutated residues as their side chains directly interact with pyrophosphate moiety of NADH. Fig 6 is shows the dihedral angle distribution of mutated residues involved in interactions with NADH. All the estimated dihedral angles are observed to be highly scattered across a window of 200° to -200°. The side chain distributions of mutated residues show large deviations from the corresponding WT residues. Only, Thr47 show side chain distribution similar to Ile47 throughout the simulation. On the other hand, Thr16 shows side chain distribution similar to Ile16 till 60ns of simulations. Similarly, Val21 also shows side chain distribution corresponding to Ile21 from 20ns to 35ns. Among the mutated residues, Ala94 shows large deviation from the corresponding residue Ser94. The smaller side chain of Ala94 allows more conformational flexibility to side chain due to diminished interactions with NADH. Unlike Ser94, side chain of the mutated residue Pro95 shows little deviation from the Ile95. Thus, it is clear from the discussion that the mutated residues with smaller side chain show deviation in side chain dihedral angle distribution as compared to the corresponding residues in WT. The space created due to side chain difference of WT and MT residues can be accounted for reduced InhA-NADH interactions which increases the conformational flexibility of NADH.

**Fig 7:**
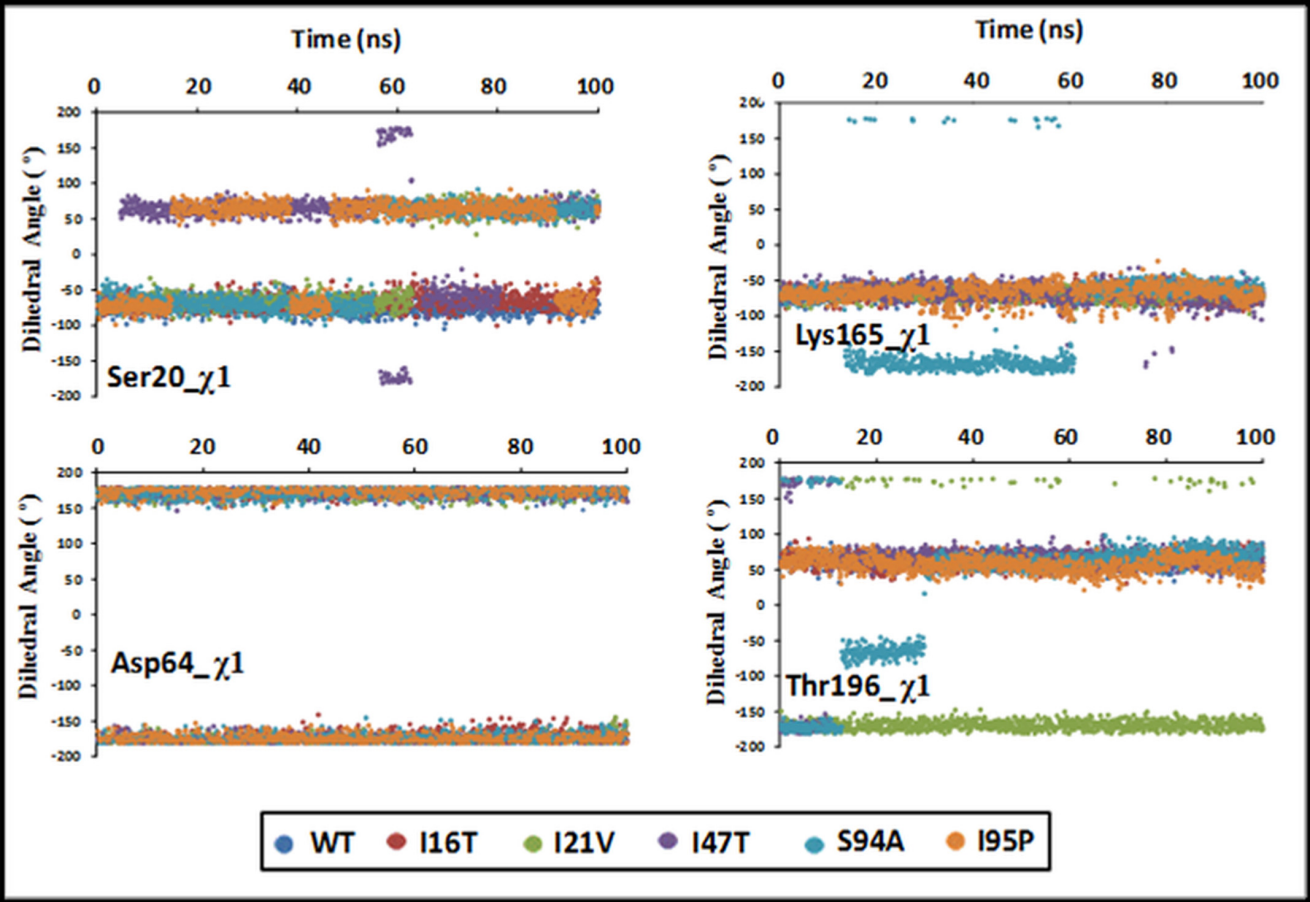
The side chain χ1 dihedral angle distribution of residues involved in NADH at DBS Of WT and MTs.

In addition to mutated residues, other binding residues around pyrophosphate moiety of NADH are also expected to face conformational changes due to distorted InhA-NADH interactions due to mutations. The χ1 dihedral angle distribution for the residue Ser20 is found scattered within a window of -100° to 100° which shows inconsistent interactions with NADH (Fig 7). In contrast to this, dihedral angle distribution for the residues Asp64, Lys165 and Thr196 is observed to be consistent. The χ1 angle distribution for the residue Asp64 is bifurcated into the two rows at about 150° and -150°. However, the H-bond with NADH is still supposed to be maintained as the rotation of side chain by this range of angle will restore the H-bond with another oxygen atom of carboxyl group.

The χ1 dihedral angle for Lys165 is observed consistent within the deviation of ~50° for all the mutants except S94A. Similarly, the χ1 dihedral angle for Thr196 is also observed very consistent for the WT and MTs within the deviation of ~50° except for I21V and S94A. Moreover, as per the comparison of χ1 among the studied residues, Asp64 is found to be more consistent than the rest of the residues. Overall, differences in dihedral angles of active site residues and NADH suggest weakening of InhA-NADH interactions which can be verified by H-bond analysis.

#### H-bond interactions between InhA-NADH in WT and MTs

The conformational changes in the side chain of binding site residues, with progress of the simulations, indicate weakening interactions with NADH. Further, to validate the deduced hypothesis, H-bond occupancy is estimated which is defined as the number of frames with H-bond for particular atom pair to the total number of frame produced in the simulation. H-bond occupancy obtained for residue-NADH interaction with a minimum cut-off of 40% is shown in Table 3 Only, six residues are observed to makes interactions with NADH in the studied complexes as compared to nine residues in crystal structures. The residues Asp64, Ile194 and Thr196 are observed to make the H-bond interactions with NADH with H-bond occupancy > 40%. However, residues Ser20 and Ile21 are observed to make H-bond interactions with NADH in WT and I21V only. Despite the mutation, Val21 makes H-bond interactions with NADH similar to Ile21. It shows that the change in the side chain of residue due to mutation does not bring change in the interaction as the backbone participates in H-bond interactions in these residues. In contrast to Ser20 and Ile21, Val65 is observed to make H-bond interactions with NADH in I47T and I95P only.

**Table 3:** Summary of H-bond interactions between NADH and InhA.

Residue	Residue atom	NADH atom	H-bond occupancy (%)					
			WT	I16T	I21V	I47T	S94A	I95P
Ser20	HG	O2	68	-	56	-	-	-
Ile21	NH	O9	42	-	58	-	-	-
Asp64	OD1	H65	-	41	46	40	-	40
	OD2	H65	-	-	-	-	-	-
	OD1/OD2	H65	49	-	-	-	56	-
Val65	NH	N4	-	-	-	47	-	45
Ile194	NH	O1	-	-	40	-	-	-
	NH	O14	62	62	-	51	53	55
	CO	H86	19	-	32	-	-	-
Thr196	HG1	O8	98	89	79	-	45	87

In context to high flexibility of pyrophosphate moiety, it is interesting to note that residues Ser20 and Ile21 are observed to maintain H-bond interaction with NADH in WT and I21V. It probably indicates the better stability of the pyrophosphate in WT and I21V as compared to other structures. Further, the detailed analysis of H-bonding within the NADH revealed the two intra-molecular H-bond interactions within NADH in WT and I21V. These interactions are observed between O2 and O5, and O8 and N7 atoms of NADH which can be accounted for the acquired rigidity of the pyrophosphate moiety. However, these residues do not make H-bond interactions with pyrophosphate moiety in I16T, I47T, S94A and I95P. It leads to comparatively better flexibility of NADH conformation in these structures as compared to WT and I21V.

**Fig 8:**
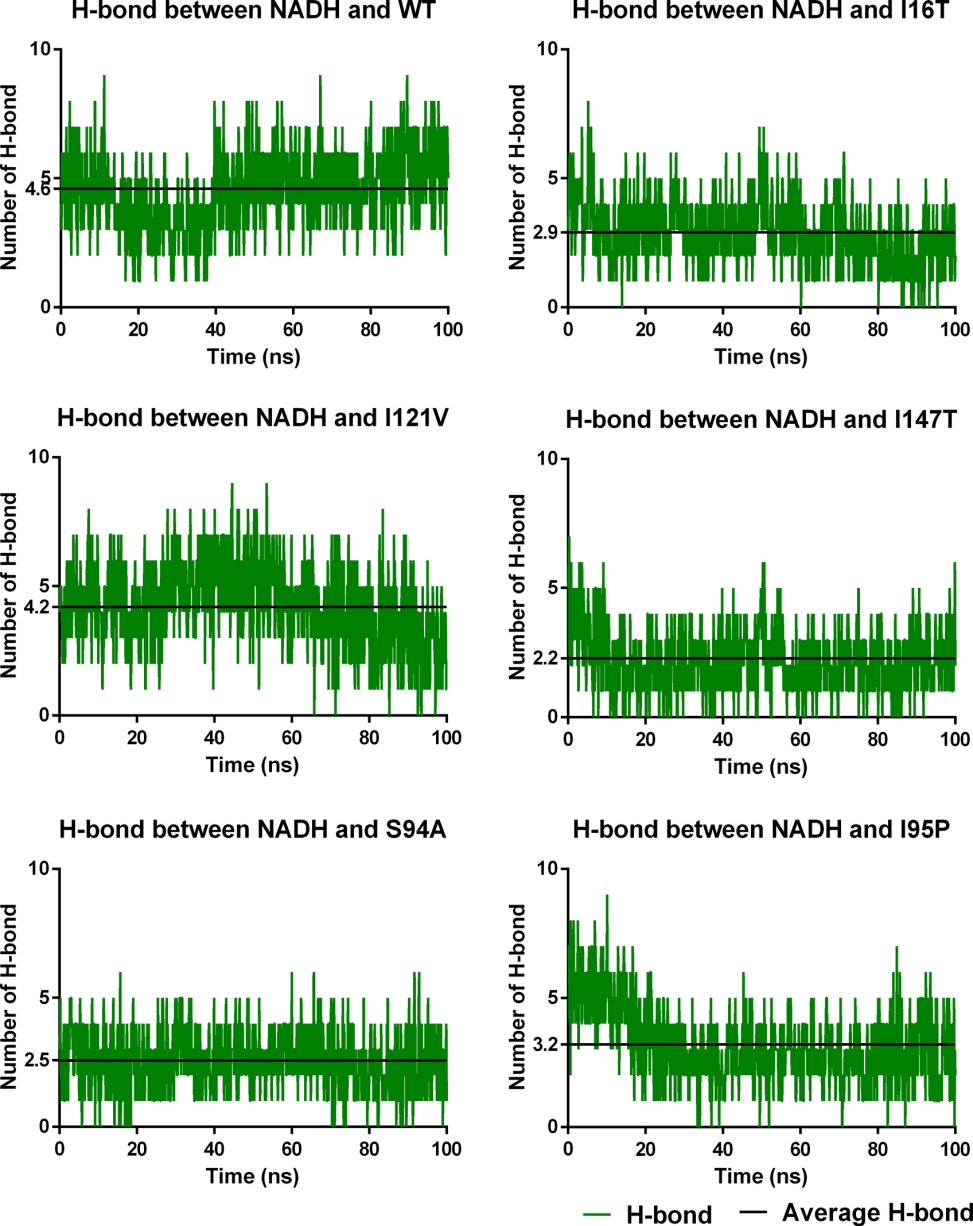
Average H-bond interactions of NADH with residues in WT and MTs.

It also indicates the comparatively better binding affinity of NADH in WT and I21V as compared to other complexes. Similarly, average H-bond analysis throughout the trajectory also indicates presence of ~5 H-bonds in WT (Fig 8). It is followed by the I21V which shows about 4 H-bonds throughout the simulations. On the other hand, I95P shows about 3 H-bond interactions while I16T and S94A show ≤3 H-bonds interactions between binding site residues and NADH. Among all studied complexes, Only I47T shows about ~2 H-bond interactions with NADH. These results are consistent with H-bond occupancy analysis which also shows better H-bond interactions in WT and I21V as compared to other complexes. Therefore, comparatively good binding energy of NADH is expected in WT. Further, it is expected to continue on with following sequence for NADH binding energy: I21V, I95P, I16T, S94A and I47T.

#### Water mediated H-bond interactions in WT and MTs

The water mediated H-bonds are also monitored in the WT and MTs throughout the 100ns simulation. Table 4 shows the occupancy of water mediated and direct H-bond to the NADH. The six crystal water molecules (WAT271, WAT272, WAT273, WAT274, WAT275 and WAT283) are observed to interact with NADH in WT and MTs. It is interesting to note that only the WAT271 is observed to maintain direct H-bond interactions in the WT and MTs except the I47T.

**Table 4:** Summary of water mediated and direct H-bond interactions in WT and MTs.

WT					I16T				
Crystal water	NADH atom	Water atom	Residue atom	H-bond occupancy (%)	Crystal water	NAD atom	Water atom	Residue atom	H-bond occupancy (%)
WAT271	O9	H1		89	WAT271	O9	H1		91
		H2	CO (Gly14)	36			H2	CO (Gly14)	46
		O	NH (Ala22)	29			O	NH (Ala22)	45
		O	HG (Ser94)	39	WAT274	O9	H1/H2		64
WAT272	N5/O6	H1/H2		78			O	HG (Ser20)	50
WAT274	O2/O9	H1/H2		46			H1/H2	CO (Gly14)	28
**I21V**					S94A				
WAT271	O9	H1		95	WAT271	O9	H1/H2		55
		H2	CO (Gly14)	49			H1/H2	CO (Gly14)	35
		O	NH (Ala22)	40			O	NH (Ala22)	32
		O	HG (Ser94)	47	WAT272	N5/O6	H1/H2		58
WAT283	O1/O8	H1/H2		42	WAT274	O5/ O9	H1/H2		61
							O	HG(Ser20)	41
I47T					**I95P**				
WAT6120	N5	H1/H2		53	WAT271	O2/O9	H1/H2		88
		H1/H2	CO (Leu63)	31			H1	CO (Gly14)	26
							O	NH (Ala22)	29
					WAT272	O6	H1/H2		75
							H1/H2	CO(Ile15)	83
					WAT273	N5	H1		55

**Fig 9:**
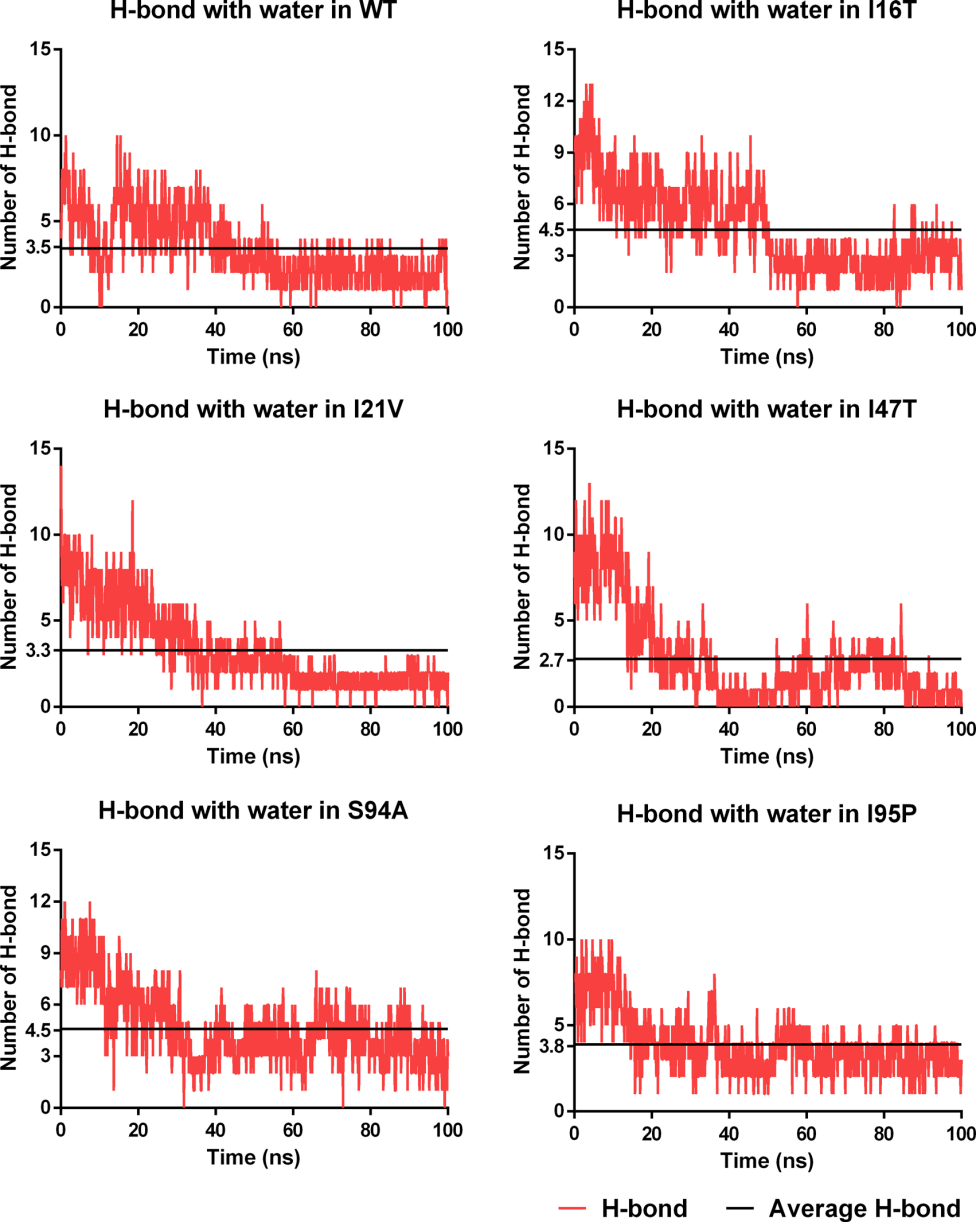
Average H-bond interactions of NADH with water in WT and MTs.

In addition to this, WAT271 also maintains the water mediated H-bond interactions through residues Gly14, Ala22 and Ser94 in WT and I21V. However, it shows interactions with residues Gly14 and Ala22 in I16T, S94A and I95P. WAT272 and WAT274 are also observed to make the H-bond with NADH in WT, S94A and I95P, and WT, I16T and S94A respectively. It is interesting to note that most of the water molecules are observed with inconsistent interactions with NADH. Moreover, with the progress of the simulations to 100ns, some new water molecules like WAT273, WAT274, WAT283 and WAT6120 are observed in the DBS. These water molecules are also found to have water mediated interactions with NADH. The analysis of the water mediated H-bond interactions demonstrates the significance of water molecules in stabilizing the NADH binding conformation in WT and MTs. It also shows the importance of WAT271 as a crucial water molecule involved in the InhA-NADH interaction with H-bond occupancy ≥55% in WT and MTs. However, with the progress of simulations, direct and water mediated interactions with NADH are drastically reduced which can be demonstrated by average H-bond analysis between NADH and water molecules. Fig 9 shows the average H-bond analysis where initial phase of trajectory shows about ~10 H-bond interactions with NADH. It is consistent with observed H-bond interactions in crystal structures. With progress of simulations, maximum average H-bond interactions are found to be about ~4 for I16T, S94A and I95P. However, these interactions are reduced to about ~3 for WT and I21V. Minimum interactions between NADH and water molecules are observed for I47T. Also, a similar pattern is observed for average H-bond interactions for last 20ns of trajectory where I16T, S94A and I95P are found with ~3 H-bond interactions with NADH while WT and I21V are observed with ~2 H-bond interactions. I47T is observed with ~1 H-bond with NADH.

These observations clearly depict that the overall interactions with NADH are reduced due to mutations. WT is fairly observed to make maximum interactions with NADH including water mediated and direct H-bond interactions. It is followed by I21V which shows about 6 interactions with NADH. Similarly, I95P also show about ~6 H-bond interactions with NADH. It is followed by I16T and S94A with 5 interactions while I47T show minimum ~3 interactions with NADH. Therefore, binding affinity of NADH towards WT and MTs is also expected in the similar sequence which can be verified by calculating binding free energy.

#### Binding free energy calculations for NADH

The comparison of the H-bond occupancy generally demonstrates the difference in the percentage of particular H-bond interaction among the trajectories produced in the MD simulations. However, it does not illustrate the stability of these interactions till the end of the simulations. Figs 8 and 9 show that interaction of NADH with residues gradually decreasing till the end of the simulations. Therefore, average of binding free energy for last 20ns of simulations can give a better estimate of binding affinity of NADH towards WT and MTs (Table 5). As per the earlier discussion on interaction of NADH with residues, WT is observed with higher binding free energy (ΔG_total_, -68.53Kcal/mol) for NADH. It is followed by I21V (ΔG_total_, -54.73Kcal/mol). It is consistent with observed H-bond interactions with NADH where WT is found with about ~7 H-bond integrations while about ~6 H-bond interactions are estimated for I21V. Despite the same number of H-bond interactions, I95P shows comparatively less binding free energy (ΔG_total_, -50.60 Kcal/mol) than I21V. Extending the comparison to I16T and S94A, only 5 H-bond interactions are observed with NADH which leads to a binding free energy of about -45kcal/mol. I47T shows the lowest binding free energy for NADH (ΔG_total_, -37.78 Kcal/mol) among the studied complexes which is consistent with observed interactions.

**Table 5:** Estimated binding free energy (kcal/mol) of NADH in WT and MTs.

Energy components	WT	I16T	I21V	I47T	S94A	I95P
**Δ*E***_**vdW**_	-78.38	-79.27	-78.84	-73.64	-75.05	-80.17
**Δ*E***_**elec**_	-148.43	-58.93	-93.08	-72.76	-86.83	-109.56
**Δ*G***_**polar,solvation**_	168.09	101.90	126.73	120.68	125.58	148.58
**Δ*G***_**nonpolar,solvation**_	-9.80	-9.30	-9.54	-9.05	-8.99	-9.51
**Δ*G***_***total***_	-68.53±6.15	-45.61±6.02	-54.73±4.79	-37.78±4.88	-45.30±4.76	-50.60±4.57
* **K**_**d**_ **(μM)**	0.57 ± 0.04	5.95 ± 0.33	13.90 ± 1.70	85.10 ± 6.30	36.40 ± 3.00	> 100
^**#**^ **K**_**d**_ **(μM)**	2.50	-	14.90	32.00	23.00	-

*Basso *et al*., 1998.

^#^ Oliveira *et al*., 2006.

Thus, the following order is obtained by comparing the binding free energy for NADH: WT > I21V > I95P > I16T > S94A > I47T. It is consistent with the observed interactions pattern. In addition to this, a high binding free energy for NADH is also in agreement with reported low dissociation constant for NADH in WT (Table 5). In contrast to this, higher dissociation constants are reported for NADH in MTs which further verifies the estimated binding affinity of NADH against MTs. It simply implies that the observed results are reasonably in agreement with the reported experimental results. In addition, it depicts the usefulness of the methodology to find the molecular details for the drug resistance phenomenon. Further, per residue energy decomposition analysis can provide a quantitative difference in NADH binding with respect to mutated residues.

#### Per residue decomposition of binding free energy

To get a deeper insight into the influence of mutations, interactions are analyzed on the basis of free energy decomposition to find the per residue contribution in the NADH binding (Table 6). The majority of the active site residues in WT show highest contribution to the NADH binding as compared to MTs. I16T, I21V, S94A and I95P to show the influence of mutation as the binding energies of the mutated residues are observed to decrease by -3.06, -1.56, -2.17 and -2.01kcal/mol respectively. In case of I16T and S94A, a low binding energy contribution by mutated residues can be accounted due to a change in the non-polar residue into polar and vice versa. It is supposed to disturb the NADH binding due to change in the nature of interacting residues. However, in case of I21V, similar interactions are maintained by the mutated residue Val21 and the small difference in energy is accounted due to reduced length of side chain only. Similarly, I95P shows the large difference in the side chain of mutated residue which is accounted for reduced interactions with NADH.

**Table 6:** Per residue energy contribution to NADH binding in WT and MTs (kcal/mol).

Residue	WT	I16T	ΔΔG	I21V	ΔΔG	I47T	ΔΔG	S94A	ΔΔG	I95P	ΔΔG
Ile16	-3.49	-0.43	-3.06	-2.66	-0.83	-1.37	-2.12	-0.69	-2.80	-1.56	-1.93
Ser20	-7.02	-0.72	-6.30	-3.68	-3.34	-0.33	-6.69	-0.75	-6.27	-0.3	-6.72
Ile21	-6.05	-3.06	-2.99	-4.49	-1.56	-1.21	-4.84	-2.28	-3.77	-1.50	-4.55
Ile47	-0.19	-0.12	-0.07	-0.17	-0.02	-0.01	-0.18	-0.10	-0.09	-0.13	-0.06
Asp64	-2.00	-2.59	0.59	-2.06	0.06	-1.85	-0.15	-2.97	0.97	-2.25	0.25
Val65	-2.36	-1.90	-0.46	-2.04	-0.32	-2.13	-0.23	-1.67	-0.69	-2.16	-0.20
Ser94	-3.13	-1.53	-1.60	-1.19	-1.94	-2.76	-0.37	-0.96	-2.17	-0.76	-2.37
Ile95	-3.70	-6.23	2.53	-4.57	0.87	-5.01	1.31	-5.39	1.69	-0.69	-3.01
Gly96	-1.15	-1.62	0.47	-1.82	0.67	-3.03	1.88	-1.67	0.52	-3.02	1.87
Lys165	-1.02	1.34	-2.36	1.41	-2.43	0.70	-1.72	-2.44	1.42	0.49	-1.51
Ile194	-3.16	-2.54	-0.62	-3.67	0.51	-2.91	-0.25	-1.89	-1.27	-3.23	0.07
Thr196	-6.40	-4.04	-2.36	-3.03	-3.37	-3.67	-2.73	-4.42	-1.98	-5.75	-0.65

ΔΔG = binding free energy difference between respective residues in WT and MTs.

However, I47T does not show the direct effect of mutation as compared to the respective residues in the WT as it is located 6Å away from the NADH binding site. Therefore, mutated residue in I47T does not show its effect on NADH binding. Moreover, apart from the mutated residues, it is important to note that the binding energy of most of the residues is also observed to decrease in the MTs except for a few residues. It shows that a single mutation is sufficient to disturb InhA-NADH binding balance which is reflected as reduced binding energy contribution of other WT residues in the MTs.
